# Disease‐Associated Risk Variants and Expression Levels of the lncRNA, CDKN2B‐AS1, Are Associated With the Progression of HCC

**DOI:** 10.1111/jcmm.70496

**Published:** 2025-03-19

**Authors:** Kuan‐Chun Hsueh, Hsiang‐Lin Lee, Kuo‐Hao Ho, Lun‐Ching Chang, Shun‐Fa Yang, Ming‐Hsien Chien

**Affiliations:** ^1^ Division of General Surgery, Department of Surgery Tungs' Taichung Metroharbor Hospital Taichung Taiwan; ^2^ Department of Post‐Baccalaureate Medicine College of Medicine, National Chung Hsing University Taichung Taiwan; ^3^ School of Medicine Chung Shan Medical University Taichung Taiwan; ^4^ Department of Surgery Chung Shan Medical University Hospital Taichung Taiwan; ^5^ Graduate Institute of Clinical Medicine, College of Medicine Taipei Medical University Taipei Taiwan; ^6^ Department of Mathematics and Statistics Florida Atlantic University Boca Raton Florida USA; ^7^ Institute of Medicine, Chung Shan Medical University Taichung Taiwan; ^8^ Department of Medical Research Chung Shan Medical University Hospital Taichung Taiwan; ^9^ Pulmonary Research Center, Wan Fang Hospital Taipei Medical University Taipei Taiwan; ^10^ Traditional Herbal Medicine Research Center Taipei Medical University Hospital Taipei Taiwan; ^11^ TMU Research Center of Cancer Translational Medicine Taipei Medical University Taipei Taiwan

**Keywords:** clinicopathologic progression, cyclin‐dependent kinase inhibitor 2B antisense RNA 1, hepatocellular carcinoma, long non‐coding RNA, single‐nucleotide polymorphism, susceptibility

## Abstract

The most susceptible loci of hepatocellular carcinoma (HCC) identified by genome‐wide association studies are located in non‐coding regions. The antisense non‐coding RNA at the INK4 locus (ANRIL), also known as cyclin‐dependent kinase inhibitor 2B antisense RNA 1 (CDKN2B‐AS1), is a long non‐coding (lnc)RNA situated within and antisense to genes encoding CDKN2A/B on chromosome 9p21.3. Single‐nucleotide polymorphisms (SNPs) within CDKN2B‐AS1 are associated with several cancer types, but their impacts on HCC remain unclear. In this study, we investigated the effects of CDKN2B‐AS1 SNPs on both the susceptibility to HCC and its clinicopathological development. Five CDKN2B‐AS1 SNP loci—rs564398 (T/C), rs1333048 (A/C), rs1537373 (G/T), rs2151280 (A/G) and rs8181047 (G/A)—were analysed using a TaqMan allelic discrimination assay for genotyping in a cohort of 810 HCC patients and 1190 healthy controls. Under the dominant model, HCC patients with at least one minor C‐allele of rs564398 showed a lower risk of liver cirrhosis (odds ratio (OR) = 0.677). Additionally, HCC patients with the GT + TT genotype of rs1537373 had a reduced risk of developing large tumours (T3 + T4) and advanced clinical stages (III/IV), particularly in the male population (OR = 0.644 and 0.679). Furthermore, data from The Cancer Genome Atlas revealed that CDKN2B‐AS1 expression levels were elevated in HCC tissues compared to normal tissues and were correlated with advanced T stages, high histological grades and poor prognoses. Our findings suggest that CDKN2B‐AS1 levels and its polymorphic variants at rs564398 and rs1537373 may influence the clinicopathological development and progression of HCC in a Taiwanese population.

## 1 Introduction

Liver cancer is the third leading cause of cancer‐related deaths and the sixth most common cancer worldwide [1]. In Taiwan, it ranks as the second leading cause of cancer fatalities [2]. Hepatocellular carcinoma (HCC), a primary liver tumour, makes up over 90% of primary liver tumours. Early‐stage HCC can be effectively treated with liver transplantation or curative surgery. However, treatment options are still limited for advanced stages of the disease [3]. Identifying risk factors is therefore crucial for developing early prevention strategies and enhancing our understanding of HCC pathogenesis. Multiple risk factors are associated with the development of HCC, including aflatoxin exposure, non‐alcoholic fatty liver disease resulting from obesity, cigarette and alcohol consumption and hepatitis B or C virus (HBV or HCV) infection [4, 5]. In addition to these environmental factors, various genetic and epigenetic variations have also been linked to HCC [6].

Genome‐wide association studies (GWASs) have identified hundreds of single‐nucleotide polymorphisms (SNPs) linked to an increased risk of major human cancers [7]. GWAS results indicate that only a very small portion of cancer‐associated SNPs are found in protein‐coding regions, while the majority (> 90%) are located in non‐coding (nc) regions [8]. In recent years, long non‐coding RNAs (lncRNAs), along with circular RNAs (circRNAs) and microRNAs(miRNAs), have become the most intensively studied non‐coding RNAs in cancers, including HCC [9, 10]. LncRNAs, a type of ncRNA longer than 200 bp, have been identified as oncogenes or tumour suppressors. They play roles in various processes of tumour progression, including uncontrollable proliferation, therapeutic resistance and metastasis [11, 12]. Growing evidence indicates that gene polymorphisms in lncRNAs are also linked to cancer risks [13] by affecting the activity or expression of lncRNAs or modulating their interactions with other biomolecules [14]. For example, the rs9914618 SNP in the lncRNA, LINC00673, was associated with an increased susceptibility to HCC in a Taiwanese population [15]. Additionally, the rs11655237 variant in LINC00673 was identified as a risk locus for pancreatic cancer by creating a binding site for miR‐1231 [16].

The lncRNA, cyclin‐dependent kinase inhibitor 2B antisense RNA 1 (CDKN2B‐AS1), also known as ANRIL (antisense non‐coding RNA at the INK4 locus), was identified to reside in a genomic hotspot at chromosome 9p21.3 [17]. This region is well known for its association with various human cancers [18, 19]. CDKN2B‐AS1 upregulation was observed in many cancer types, including HCC [20], and is recognised as a crucial factor in regulating cancer cell proliferation, migration, invasion and metastasis through various mechanisms. For instance, CDKN2B‐AS1 can act as a competing endogenous (ce)RNA, forming hybrid complexes with miR‐122‐5p or miR‐199a‐5p, thereby promoting HCC growth and metastasis [21, 22]. Additionally, CDKN2B‐AS1 was shown to regulate HCC proliferation by epigenetically repressing LF2 transcription through binding with polycomb repressive complex 2 (PRC2) [20]. Regarding the impact of CDKN2B‐AS1 SNPs on cancer, 34 SNPs have been identified as being associated with a higher risk of developing various types of cancer, including lung, breast, gastric, thyroid, brain, endometrial, cervical, prostate, ovarian, head/neck and pancreatic cancers, among others [19]. Additionally, CDKN2B‐AS1 SNPs were correlated with larger tumour sizes (rs11333048) in thyroid [23] and oral cancers [24], as well as with higher TNM (tumour, node, metastasis) stages and increased metastasis risk (rs3217992) in bone cancer [25]. Furthermore, CDKN2B‐AS1 rs1333049 and rs2383207 SNPs were respectively reported to be correlated with overall survival (OS) in patients with head/neck and breast cancers [26, 27]. Although several studies investigated the clinical significance and functional role of CDKN2B‐AS1 in HCC, the impacts of CDKN2B‐AS1 genetic variants on HCC remain unclear. Therefore, we conducted a case–control study in a Taiwanese population to identify the roles of CDKN2B‐AS1 SNPs in the risks and clinical characteristics of HCC.

## 2 Materials and Methods

### 2.1 Study Populations and Ethics

In this case–control study, blood samples from 563 male and 247 female HCC patients were collected from Chung Shan Medical University Hospital (Taichung, Taiwan) and the National Biobank Consortium of Taiwan (NBCT). All HCC patients were pathologically confirmed and clinically staged according to the TNM staging system of the American Joint Committee on Cancer (AJCC). In total, 1190 healthy controls, matched for gender, age and ethnicity, were randomly selected from the Taiwan Biobank Project. Information about their smoking and alcohol consumption history was obtained from all participants through interviewer‐administered questionnaires. Written informed consent was obtained from each participant before collecting venous blood, and the investigation protocol was approved by the Institutional Review Board (IRB no. CS2‐19133) of Chung Shan Medical University Hospital.

### 2.2 Genomic DNA Extraction From Blood

Whole blood samples were collected in tubes containing ethylenediaminetetraacetic acid (EDTA). Following centrifugation, genomic DNA was isolated from buffy coats using a QIAamp DNA Blood Mini Kit (Qiagen, Valencia, CA, USA) according to the manufacturer's instructions. Before using the isolated DNA as templates for a polymerase chain reaction (PCR), the DNA quality was assessed with a Nanodrop‐2000 spectrophotometer (Thermo Scientific, Waltham, MA, USA).

### 2.3 Selection and Genotyping of CDKN2B‐AS1 SNPs

In this study, we selected five SNPs (rs564398, rs1333048, rs1537373, rs2151280 and rs8181047) because their minor allele frequencies were more than 5%, and these SNPs have been significantly associated with susceptibility or traits such as tumour size in various cancers [19, 24, 28]. Allelic discrimination of the CDKN2B‐AS1 SNPs of rs564398, rs1333048, rs1537373, rs2151280 and rs8181047 was conducted using a TaqMan SNP Genotyping Assay, utilising the ABI StepOnePlus Real‐Time PCR System (Applied Biosystems, Foster City, CA, USA). The final results were analysed using SDS v. 3.0 software (Applied Biosystems).

### 2.4 Bioinformatics Analysis

RNA sequencing (RNA‐Seq) and clinical data of liver HCC (LIHC) patients were retrieved from UCSC Xena (https://xena.ucsc.edu/). RNA‐Seq data were normalised using RNA‐Seq by Expectation–Maximisation (RSEM) and were log2‐transformed. For two‐group comparisons, a Wilcoxon rank‐sum test was conducted to evaluate statistical differences, such as tumour versus normal tissues, lymph node involvement and metastasis comparisons. For TNM staging and tumour grade, which have more than two groups, a Kruskal–Wallis test with post hoc Dunn's test was performed to evaluate the significance. For the survival analysis, a log‐rank test was used to assess survival differences. For the pathway analysis, a gene set enrichment analysis (GSEA) was conducted. Correlation coefficients of each gene with CDKN2BAS were evaluated using Pearson correlations. Subsequently, genes were ranked based on these correlation coefficients. Then, gene sets from Hallmark and Kyoto Encyclopedia of Genes and Genomes (KEGG) were utilised to perform the GSEA. Pathways with a false discovery rate (FDR) of < 0.01 and a normalised enrichment score (NES) of > 0 are shown.

### 2.5 Statistical Analysis

Relationships between genotype frequencies and the risk or clinicopathologic features of HCC were assessed through multiple logistic regression models, adjusted for potential confounding variables. Statistical analyses were conducted using the SAS software program (v9.4, 2013; SAS Institute, Cary, NC, USA), with statistical significance defined as *p* < 0.05.

## 3 Results

### 3.1 Demographic Characteristics of Recruited Subjects

Table 1 presents the demographic characteristics of recruited subjects. The study group consisted of 810 pathologically confirmed HCC patients (563 males and 247 females) and 1190 cancer‐free controls (830 males and 360 females). No significant differences were observed between HCC patients and healthy controls regarding age distributions (< 60 and ≥ 60 years, *p* = 0.308), gender (*p* = 0.908) or smoking status (*p* = 0.372). Consistent with an HCC population reported from Taiwan [29], our recruited HCC patients had significantly higher frequencies of alcohol consumption (33.8% vs. 14.0%; *p* < 0.001), hepatitis B surface antigen (HBsAg) positivity (33.7% vs. 12.2%; *p* < 0.001) and anti‐HCV antibody positivity (33.3% vs. 4.5%; *p* < 0.001) compared to controls. Higher proportions of HCC patients were diagnosed at early clinical stages (73.5% at stages I + II) and early T stages (74.3% at T1 + T2). Additionally, 59.3% had liver cirrhosis, while 97.3% had no lymph node involvement (N0), 95.4% had no distant metastasis (M0) and 65.2% showed no vascular invasion.

**TABLE 1:** Distributions of demographical characteristics in 1190 controls and 810 patients with hepatocellular carcinoma (HCC).

Variable	Controls (*N* = 1190)	Patients (*N* = 810)	*p*
Age (years)			
< 60	478 (40.2%)	307 (37.9%)	0.308
≥ 60	712 (59.8%)	503 (62.1%)	
Gender			
Male	830 (69.7%)	563 (69.5%)	0.908
Female	360 (30.3%)	247 (30.5%)	
Cigarette smoking			
No	723 (60.8%)	476 (58.8%)	0.372
Yes	467 (39.2%)	334 (41.2%)	
Alcohol consumption			
No	1023 (86.0%)	536 (66.2%)	< 0.001*
Yes	167 (14.0%)	274 (33.8%)	
HBsAg			
Negative	1045 (87.8%)	537 (66.3%)	*p* < 0.001*
Positive	145 (12.2%)	273 (33.7%)	
Anti‐HCV			
Negative	1137 (95.5%)	540 (66.7%)	< 0.001*
Positive	53 (4.5%)	270 (33.3%)	
Stage			
I + II		595 (73.5%)	
III + IV		215 (26.5%)	
Tumour (T) status			
T1 + T2		602 (74.3%)	
T3 + T4		208 (25.7%)	
Lymph node (N) status			
N0		788 (97.3%)	
N1 + N2 + N3		22 (2.7%)	
Metastasis (M)			
M0		733 (95.4%)	
M1		37 (4.6%)	
Vascular invasion			
No		528 (65.2%)	
Yes		282 (34.8%)	
Liver cirrhosis			
Negative		330 (40.7%)	
Positive		480 (59.3%)	

Abbreviations: HBsAg, hepatitis B virus surface antigen; HCV, hepatitis C virus.

* *p* < 0.05 was accepted as statistically significant.

### 3.2 Associations Between CDKN2B‐AS1 SNPs and HCC Susceptibility

**FIGURE 1:**
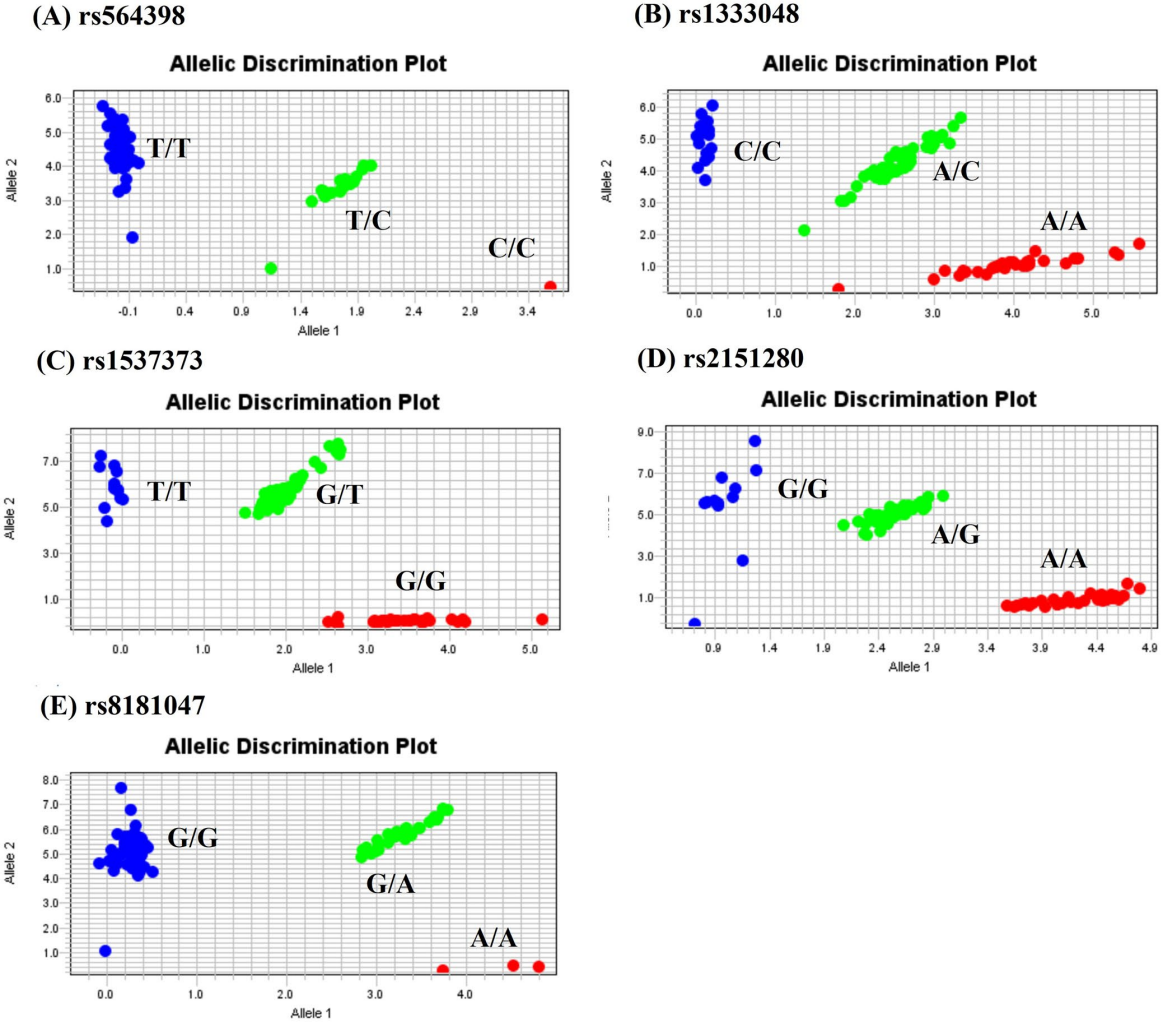
Allelic discrimination plot obtained for the CDKN2B‐AS1 SNP (A) rs564398, (B) rs1333048, (C) rs1537373, (D) rs2151280 and (E) rs8181047 using a TaqMan assay. The *x*‐ and *y*‐axes indicate the fluorescence values of the VIC and FAM dyes, respectively, while the dots are individual sample points.

To explore potential associations between CDKN2B‐AS1 SNPs and the risk of developing HCC, we analysed genotype frequencies of five SNPs (rs564398, rs1333048, rs1537373, rs2151280 and rs8181047) in our recruited population. As shown in Figure 1, the most frequently occurring alleles were heterozygous A/C for rs1333048, G/T for rs1537373 and A/G for rs2151280, while homozygous T/T was most common for rs564398 and G/G for rs8181047 (Figure 1). After adjusting for potential confounding factors, no significant correlations were found between these CDKN2B‐AS1 variants and the occurrence of HCC when comparing HCC patients and controls (Table 2). This result was consistent whether assessed using a dominant or codominant model. Moreover, we compared the data for these five CDKN2B‐AS1 SNPs using dbSNP, 1000 Genomes and the HapMap study from the National Center for Biotechnology Information database. As shown in Table 3, the allele frequencies of CDKN2B‐AS1 SNPs are consistent across these three databases and our study.

**TABLE 2:** Genotyping and allele frequency of *CDKN2B‐AS1* single nucleotide polymorphisms (SNPs) in hepatocellular carcinoma (HCC) patients and normal controls.

Variable	Controls (*N* = 1190) (%)	Patients (*N* = 810) (%)	AOR (95% CI)
**rs564398**			
TT	935 (78.6%)	638 (78.8%)	1.000 (reference)
TC	246 (20.7%)	161 (19.9%)	0.891 (0.682–1.164)
CC	9 (0.7%)	11 (1.3%)	1.871 (0.646–5.417)
TC + CC	255 (21.4%)	172 (21.2%)	0.961 (0.843–1.095)
**rs1333048**			
AA	322 (27.1%)	228 (28.1%)	1.000 (reference)
AC	592 (49.7%)	387 (47.8%)	0.859 (0.667–1.106)
CC	276 (23.2%)	195 (24.1%)	0.946 (0.703–1.272)
AC + CC	868 (72.9%)	582 (71.9%)	0.942 (0.836–1.060)
**rs1537373**			
GG	491 (41.3%)	344 (42.5%)	1.000 (reference)
GT	546 (45.9%)	369 (45.5%)	0.942 (0.750–1.183)
TT	153 (12.8%)	97 (12.0%)	0.988 (0.703–1.389)
GT + TT	699 (58.7%)	466 (57.5%)	0.976 (0.876–1.087)
**rs2151280**			
AA	526 (44.2%)	349 (43.1%)	1.000 (reference)
AG	529 (44.5%)	364 (44.9%)	1.067 (0.851–1.338)
GG	135 (11.3%)	97 (12.0%)	1.107 (0.778–1.575)
AG + GG	664 (55.8%)	461 (56.9%)	1.037 (0.931–1.154)
**rs8181047**			
GG	896 (75.3%)	622 (76.8%)	1.000 (reference)
GA	280 (23.5%)	172 (21.2%)	0.878 (0.678–1.136)
AA	14 (1.2%)	16 (2.0%)	1.531 (0.643–3.646)
GA + AA	294 (24.7%)	188 (23.2%)	0.954 (0.841–1.081)

*Note:* Adjusted for the effects of age, gender, cigarette smoking, alcohol drinking, hepatitis B surface antigen and anti‐hepatitis C virus.

Abbreviations: AOR, adjust odds ratio; CI, confidence interval.

**TABLE 3:** Allele frequency of *CDKN2B‐AS1* single nucleotide polymorphisms (SNPs) in a public database.

Study	Population/ethnicity	Sample size	CDKN2B‐AS1 SNPs	
			**rs564398**	
			**T**	**C**
This study	Taiwanese	1190	88.91%	11.09%
dbSNP (NCBI)	East Asian	4954	88.09%	11.91%
dbSNP (NCBI)	Asian	6938	85.62%	14.38%
1000 Genomes	South Asian	978	72.70%	27.30%
HapMap	Asian	254	91.70%	8.30%
			**rs1333048**	
			**A**	**C**
This study	Taiwanese	1190	51.93%	40.87%
dbSNP (NCBI)	East Asian	4538	51.90%	48.10%
dbSNP (NCBI)	Asian	6388	52.97%	47.03%
1000 Genomes	South Asian	978	48.40%	51.60%
HapMap	Asian	254	44.50%	55.50%
			**rs1537373**	
			**G**	**T**
This study	Taiwanese	1190	64.20%	35.80%
dbSNP (NCBI)	East Asian	166	65.70%	34.30%
dbSNP (NCBI)	Asian	192	66.10%	33.90%
1000 Genomes	South Asian	978	58.60%	41.40%
HapMap	Asian	90	74.00%	26.00%
			**rs2151280**	
			**A**	**G**
This study	Taiwanese	1190	66.43%	33.57%
dbSNP (NCBI)	East Asian	148	72.30%	27.70%
dbSNP (NCBI)	Asian	166	71.70%	28.30%
1000 Genomes	South Asian	978	61.10%	38.90%
HapMap	Asian	254	68.10%	31.90%
			**rs8181047**	
			**G**	**A**
This study	Taiwanese	1190	87.06%	12.94%
dbSNP (NCBI)	East Asian	146	89.00%	11.00%
dbSNP (NCBI)	Asian	204	87.30%	12.70%
1000Genomes	South Asian	978	75.90%	24.10%
HapMap	Asian	254	89.40%	10.60%

### 3.3 Impacts of CDKN2B‐AS1 Genetic Polymorphisms on Clinicopathologic Features of HCC Patients

We then investigated potential correlations between CDKN2B‐AS1 genetic polymorphisms and various clinicopathological features, including clinical stage, primary tumour size, lymph node and distant metastases, vascular invasion, hepatitis virus infection and liver cirrhosis among all HCC patients. As shown in Table 4, patients with at least one minor allele of rs564398 (TC and CC) had a significantly reduced risk of developing liver cirrhosis compared to those with the wild‐type (WT) genotype (TT) (odds ratio (OR), 0.677; *p* = 0.024). Additionally, HCC patients carrying at least one minor allele of rs1537373 (GT and TT) had a significantly lower risk (0.717‐fold, *p* = 0.039) of developing larger tumour sizes (T3 + T4) compared to those with the WT genotype (GG) (Table 5). Further analysis of the HCC population by sex revealed that only male HCC patients with at least one minor allele of rs1537373 had a significantly reduced risk of developing larger tumour sizes (T3 + T4) and advanced clinical stages (III + IV) (Table 6). The other three CDKN2B‐AS1 SNPs (rs1333048, rs2151280 and rs8181047) showed no significant associations with the clinicopathological features mentioned above (data not shown).

**TABLE 4:** Odds ratios (ORs) and 95% confidence intervals (CIs) of the clinical status and *CDKN2B‐AS1* rs564398 genotypic frequencies in hepatocellular carcinoma (HCC) patients.

Variable	Genotypic frequencies			
	TT (*N* = 638)	TC + CC (*N* = 172)	OR (95% CI)	*p*
Clinical stage				
Stage I/II	467 (73.2%)	128 (74.4%)	1.000	0.748
Stage III/IV	171 (26.8%)	44 (25.6%)	0.939 (0.639–1.379)	
Tumour size				
T1 + T2	471 (73.8%)	131 (76.2%)	1.000	0.533
T3 + T4	167 (26.2%)	41 (23.8%)	0.883 (0.596–1.307)	
Lymph node metastasis				
No	620 (97.2%)	168 (97.7%)	1.000	0.723
Yes	18 (2.8%)	4 (2.3%)	0.820 (0.274–2.456)	
Distant metastasis				
No	608 (95.3%)	165 (95.9%)	1.000	0.724
Yes	30 (4.7%)	7 (4.1%)	0.860 (0.371–1.193)	
Vascular invasion				
No	423 (66.3%)	105 (61.0%)	1.000	0.199
Yes	215 (33.7%)	67 (39.0%)	1.255 (0.887–1.777)	
HBsAg				
Negative	431 (67.6%)	106 (61.6%)	1.000	0.144
Positive	207 (32.4%)	66 (38.4%)	1.296 (0.914–1.838)	
Anti‐HCV				
Negative	426 (66.8%)	114 (66.3%)	1.000	0.903
Positive	212 (33.2%)	58 (33.7%)	1.022 (0.716–1.460)	
Liver cirrhosis				
Negative	247 (38.7%)	83 (48.3%)	**1.000**	**0.024** *
Positive	391 (61.3%)	89 (51.7%)	**0.677 (0.483–0.951)**	

*Note:* The ORs with their 95% CIs were estimated by logistic regression models. Bold values indicate statistical significance.

Abbreviations: HBsAg, hepatitis B surface antigen; HCV, hepatitis C virus.

* *p* < 0.05 was accepted as statistically significant.

**TABLE 5:** Odds ratios (ORs) and 95% confidence intervals (CIs) of the clinical status and *CDKN2B‐AS1* rs1537373 genotypic frequencies in hepatocellular carcinoma (HCC) patients.

Variable	Genotypic frequencies			
	GG (*N* = 344)	GT + TT (*N* = 466)	OR (95% CI)	*p*
Clinical stage				
Stage I/II	242 (70.3%)	353 (75.8%)	1.000	0.085
Stage III/IV	102 (29.7%)	113 (24.2%)	0.759 (0.555–1.039)	
Tumour size				
T1 + T2	243 (70.6%)	359 (77.0%)	**1.000**	**0.039** *
T3 + T4	101 (29.4%)	107 (23.0%)	**0.717 (0.522–0.984)**	
Lymph node metastasis				
No	339 (98.5%)	449 (96.4%)	1.000	0.058
Yes	5 (1.5%)	17 (3.6%)	2.567 (0.938–7.027)	
Distant metastasis				
No	332 (96.5%)	441 (94.6%)	1.000	0.206
Yes	12 (3.5%)	25 (5.4%)	1.568 (0.777–3.168)	
Vascular invasion				
No	225 (64.4%)	303 (65.0%)	1.000	0.909
Yes	119 (34.6%)	163 (35.0%)	1.017 (0.759–1.363)	
HBsAg				
Negative	223 (64.8%)	314 (67.4%)	1.000	0.447
Positive	121 (35.2%)	152 (32.6%)	0.892 (0.665–1.197)	
Anti‐HCV				
Negative	237 (68.9%)	303 (65.0%)	1.000	0.248
Positive	107 (31.1%)	163 (35.0%)	1.192 (0.885–1.604)	
Liver cirrhosis				
Negative	143 (41.6%)	187 (40.1%)	1.000	0.680
Positive	201 (58.4%)	279 (59.9%)	1.061 (0.800–1.409)	

*Note:* The ORs with their 95% CIs were estimated by logistic regression models. Bold values indicate statistical significance.

Abbreviations: HBsAg, hepatitis B surface antigen; HCV, hepatitis C virus.

* *p* < 0.05 was accepted as statistically significant.

**TABLE 6:** Odds ratios (ORs) and 95% confidence intervals (CIs) of the clinical status and *CDKN2B‐AS1* rs1537373 genotypic frequencies in 563 male hepatocellular carcinoma (HCC) patients.

Variable	Genotypic frequencies			
	GG (*N* = 234)	GT + TT (*N* = 329)	OR (95% CI)	*p*
Clinical stage				
Stage I/II	158 (67.5%)	248 (75.4%)	**1.000**	**0.040** *
Stage III/IV	76 (32.5%)	81 (24.6%)	**0.679 (0.468–0.984)**	
Tumour size				
T1 + T2	157 (67.1%)	250 (76.0%)	**1.000**	**0.020** *
T3 + T4	77 (32.9%)	79 (24.0%)	**0.644 (0.444–0.935)**	
Lymph node metastasis				
No	230 (98.3%)	315 (95.7%)	1.000	0.091
Yes	4 (1.7%)	14 (4.3%)	2.556 (0.830–7.865)	
Distant metastasis				
No	225 (96.2%)	309 (93.9%)	1.000	0.237
Yes	9 (3.8%)	20 (6.1%)	1.618 (0.723–3.620)	
Vascular invasion				
No	147 (62.8%)	212 (64.4%)	1.000	0.694
Yes	87 (37.2%)	117 (35.6%)	0.932 (0.658–1.321)	
HBsAg				
Negative	156 (66.7%)	219 (66.6%)	1.000	0.980
Positive	78 (33.3%)	110 (33.4%)	1.005 (0.704–1.433)	
Anti‐HCV				
Negative	173 (73.9%)	227 (69.0%)	1.000	0.203
Positive	61 (26.1%)	102 (31.0%)	1.274 (0.877–1.852)	
Liver cirrhosis				
Negative	105 (44.9%)	138 (41.9%)	1.000	0.490
Positive	129 (55.1%)	191 (58.1%)	1.127 (0.803–1.580)	

*Note:* The ORs with their 95% CIs were estimated by logistic regression models. Bold values indicate statistical significance.

Abbreviations: HBsAg, hepatitis B surface antigen; HCV, hepatitis C virus.

* *p* < 0.05 was accepted as statistically significant.

### 3.4 Elevated CDKN2B‐AS1 Levels in HCC Tissues Were Correlated With Tumour Progression and Poor Prognoses

**FIGURE 2:**
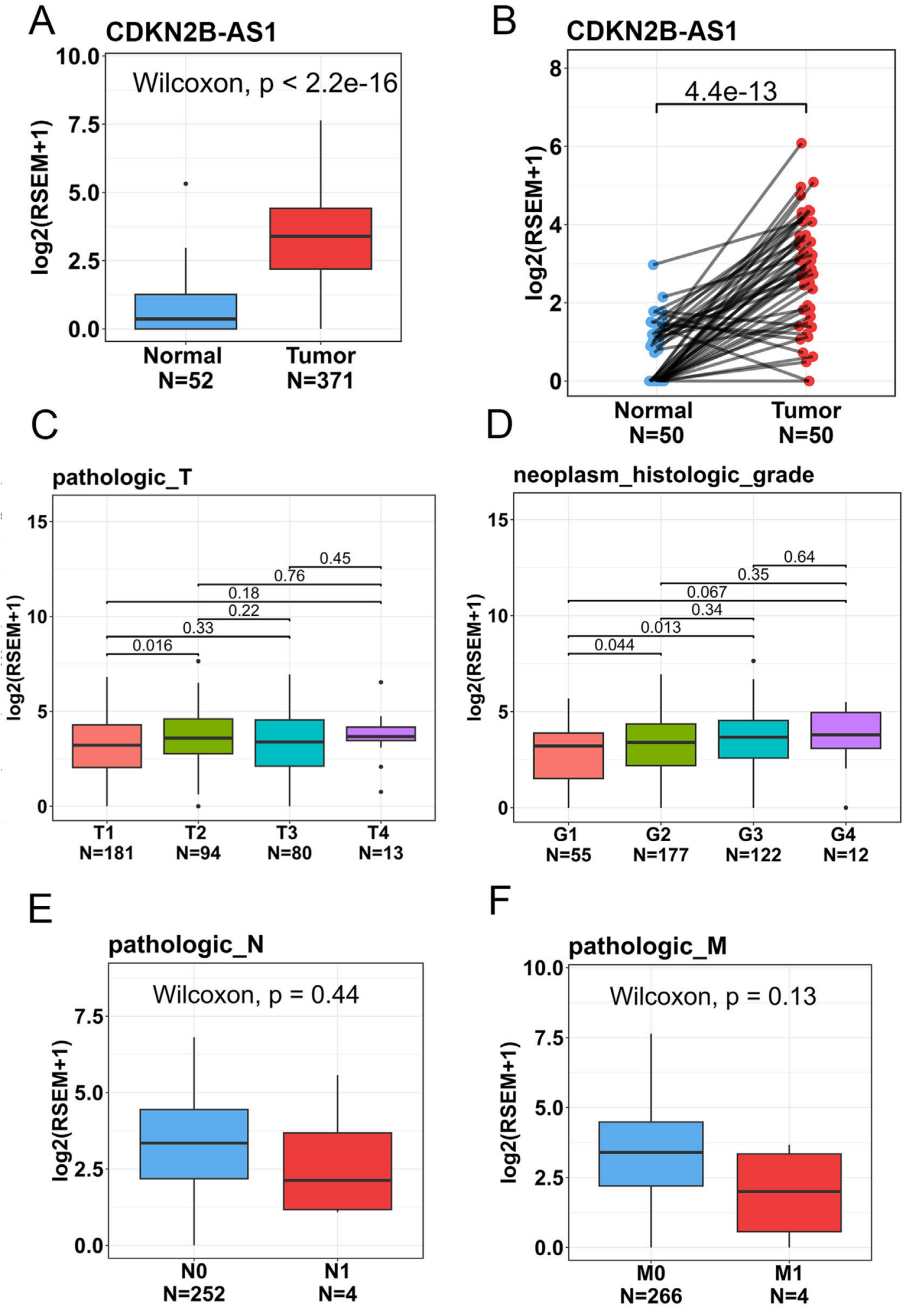
Clinical relevance of cyclin‐dependent kinase inhibitor 2B antisense RNA 1 (CDKN2B‐AS1) levels in patients with hepatocellular carcinoma (HCC) obtained from the TCGA‐liver hepatocellular carcinoma (LIHC) dataset. (A) The boxplot demonstrates that CDKN2B‐AS1 was significantly elevated in LIHC tumour tissues compared to normal tissues in TCGA‐LIHC patients. A Wilcoxon rank‐sum test was performed to evaluate statistical differences. (B) The paired dot plot indicates that CDKN2B‐AS1 expression was higher in tumour tissues compared to paired normal tissues. A paired *t*‐test was conducted to evaluate statistical differences. (C–F) CDKN2B‐AS1 expression levels in HCC from TCGA‐LIHC were compared according to the pathological T stage (C), tumour grade (D), lymph node metastasis (E) and distal metastasis (F). A Wilcoxon rank‐sum test was performed to evaluate statistical differences in two‐group comparisons. A Kruskal–Wallis test with post hoc Dunn's test was performed to evaluate the significance for more than two‐group comparisons.

**FIGURE 3:**
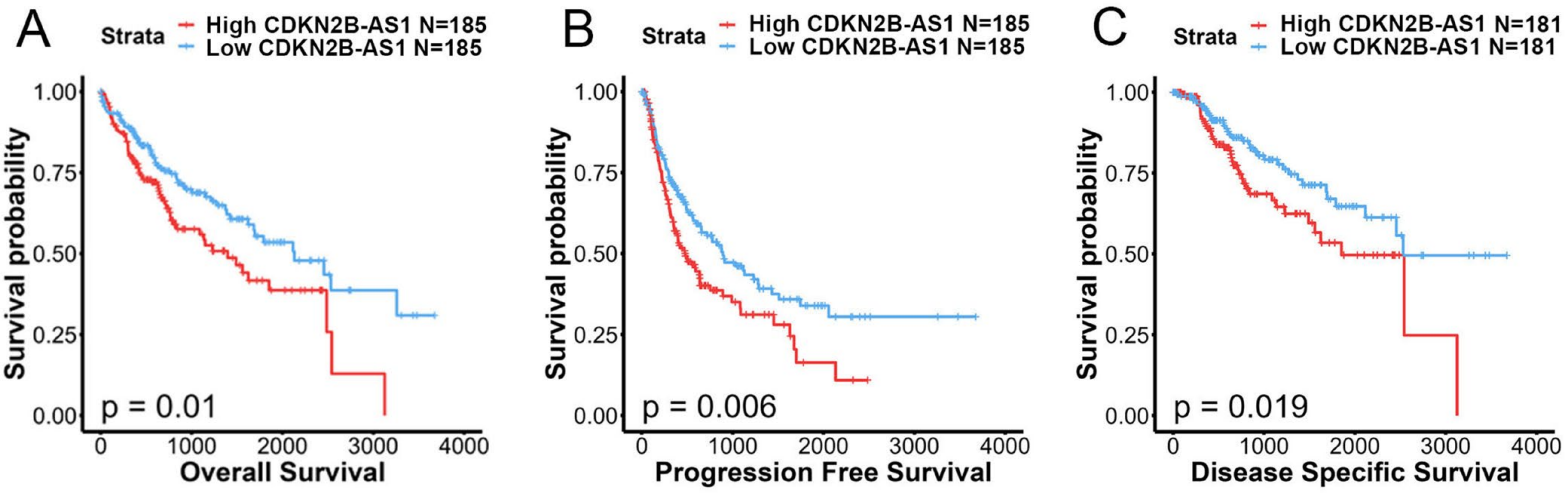
High cyclin‐dependent kinase inhibitor 2B antisense RNA 1 (CDKN2B‐AS1) expression predicts poor survival outcomes in hepatocellular carcinoma (HCC) patients. Kaplan–Meier plots show that elevated CDKN2B‐AS1 expression predicts poor outcomes for overall survival (A), progression‐free survival (B) and disease‐specific survival (C) in HCC patients. Patients with high or low expression were classified based on median expression levels of CDKN2B‐AS1. A log‐rank test was used to evaluate statistical differences (database source: TCGA‐liver HCC).

Given potential impacts of CDKN2B‐AS1 polymorphic genotypes on expression levels of CDKN2B‐AS1 [30], we further analysed correlations of CDKN2B‐AS1 expression levels with clinical significance and survival rates in HCC patients by examining cases from the TCGA‐LIHC dataset. We observed that CDKN2B‐AS1 expression levels were significantly higher in tumour tissues compared to non‐cancerous tissues (Figure 2A) and their corresponding matched normal tissues (Figure 2B). Furthermore, HCC patients with elevated CDKN2B‐AS1 transcript levels were associated with higher pathological T stages (Figure 2C) and histological grades (Figure 2D). However, no significant correlations were found between increased CDKN2B‐AS1 expression levels and lymph node involvement (Figure 2E) or distant metastases (Figure 2F), suggesting that CDKN2B‐AS1 might play a role in the process of HCC growth and development. Kaplan–Meier plots indicated that HCC patients from the TCGA‐LIHC dataset with high CDKN2B‐AS1 expression (CDKN2B‐AS1^high^) had shorter OS (Figure 3A), progression‐free survival (Figure 3B) and disease‐specific survival times (Figure 3C) compared to those with low CDKN2B‐AS1 expression (CDKN2B‐AS1^low^).

### 3.5 Analysis of Potential Molecular Mechanisms Regulated by CDKN2B‐AS1 in HCC Progression

**FIGURE 4:**
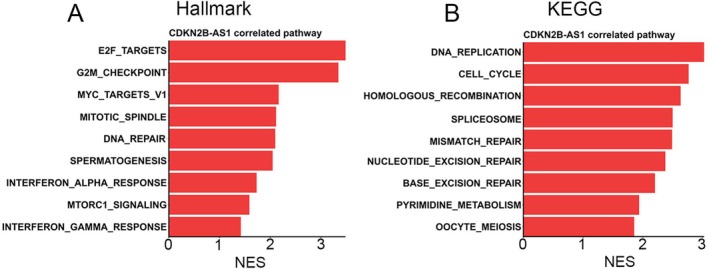
Pathways positively associated with cyclin‐dependent kinase inhibitor 2B antisense RNA 1 (CDKN2B‐AS1) in hepatocellular carcinoma (HCC) patients. Bar plots demonstrate significant signalling pathways correlated with CDKN2BAS expression in Hallmark (A) and KEGG (B) databases. A gene set enrichment analysis (GSEA) was conducted to evaluate the associations. Pathways with a false discovery rate (FDR) of < 0.01 and a normalised enrichment score (NES) of > 0 are shown.

To further elucidate the mechanism underlying CDKN2B‐AS1‐modulated HCC progression, we performed a GSEA using the TCGA‐LIHC dataset. The analysis revealed that ‘E2F targets’ and ‘G_2_M checkpoint’ were the top two Hallmark gene sets in the CDKN2B‐AS1‐high group (Figure 4A). Additionally, ‘DNA replication’ was the top pathway identified in the GSEA, followed by ‘cell cycle’, based on the KEGG database in HCC with high CDKN2B‐AS1 expression (Figure 4B). These pathway analyses suggested that CDKN2B‐AS1 may play a critical role in regulating HCC proliferation.

## 4 Discussion

HCC continues to be a leading cause of cancer‐related deaths worldwide. Despite great efforts to identify effective biomarkers for monitoring and early diagnosis of HCC, results remain inadequate. Current serum biomarkers exhibit low sensitivity and heterogeneous specificities, even when different cutoff points or combinations of multiple biomarkers are used [31]. lncRNAs, a type of ncRNA, were reported to exhibit tissue‐specific expression patterns, making them highly valuable for predicting the occurrence, progression and prognosis of various cancers, including HCC [32, 33, 34]. For example, the lncRNAs, ADAMTSL4‐AS1, AC067931 and SOCS2‐AS1, in peripheral blood mononuclear cells were identified as novel biomarkers for HBV‐associated HCC [35]. Moreover, the lncRNA, CASC2, was proven to be a better diagnostic biomarker for HCC than the alpha‐fetoprotein (AFP) routine biomarker [36]. Furthermore, Zheng et al. indicated that upregulation of the UCA1 lncRNA in the sera of HCC patients was associated with advanced TNM stages [37]. CDKN2B‐AS1, an lncRNA first identified in 2011, was recognised as a risk factor for coronary atherosclerosis and type 2 diabetes [38]. Regarding its role in HCC, Huang et al. found that CDKN2B‐AS1 targets let‐7c‐5p/NAP1L1 to promote HCC growth [39], while Li et al. discovered that CDKN2B‐AS1 targets miR‐199a‐5p/ARL2 to enhance mitochondrial function in HCC [22]. Additionally, Tao et al. demonstrated that CDKN2B‐AS1 promotes cell cycle progression and proliferation in HCC cells by targeting E2F transcription factor 1 (E2F1) [40]. Similarly, circMYBL2 was reported to enhance HCC proliferation by regulating E2F1 expression [9]. Furthermore, Aurora Kinase A (AURKA) has been identified as a key regulator of cell cycle progression in HCC [41]. Thus, further investigation is needed to explore the interplay among CDKN2B‐AS1, circMYBL2 and AURKA in the regulation of cell cycle progression in HCC. In our study, we also found that CDKN2B‐AS1 was elevated in HCC and was correlated with advanced T stages, high histologic grades and poor prognoses in HCC patients. Although the oncogenic roles of CDKN2B‐AS1 in HCC have been studied, there has been limited investigation into epidemiological aspects of HCC susceptibility and clinicopathologic characteristics conferred by genetic variants at CDKN2B‐AS1 loci.

In the present study, we observed that HCC patients with a mutant base C of rs564398 (TC + CC) had a significantly lower risk of developing liver cirrhosis under a dominant model. The rs564398 SNP, with a major allele (T) and a minor allele (C), is located in an alternative, non‐constitutive cassette second exon of the CDKN2B‐AS1 lncRNA transcriptional unit. This SNP overlaps with a putative binding site for RAS‐responsive element binding protein 1 (RREB1) [38]. RREB1, a zinc finger transcription factor, is involved in various biological processes, including fibrogenic epithelial‐to‐mesenchymal transitions (EMTs) [42]. RREB1 binding at this site likely mediates RAS‐dependent CDKN2B‐AS1 downregulation. The T allele of rs564398 was strongly correlated with CDKN2B‐AS1 downregulation in peripheral blood and melanoma cells [43, 44]. Conversely, the C allele of rs564398 was predicted to disrupt this RREB1‐binding site [38], preventing RREB1 binding and consequently CDKN2B‐AS1 downregulation. CDKN2B‐AS1 downregulation was reported to enhance liver fibrosis and hepatic stellate cell (HSC) activation [45]. Activated HSCs transform into myofibroblast‐like cells, promoting fibrosis in response to chronic liver inflammation, leading to cirrhosis and HCC [46]. Taken together, these results suggest that genetic mutations of CDKN2B‐AS1 rs564398 might influence messenger (m)RNA expression through RREB1 binding and subsequently affect the development of liver cirrhosis. Future research should further explore relationships between CDKN2B‐AS1 rs564398 SNPs and RREB1 in regulating CDKN2B‐AS1 expression in HCC.

In addition to rs564398, our results indicated that patients with the mutant base T of rs1537373 had a significantly lower risk of developing larger tumours (T3 + T4) under a dominant model (GT + TT), particularly in the male population. Zhu et al. similarly found that individuals with the mutant base T of rs1537373 had a significantly lower risk of pancreatic cancer (PC) under both codominant and dominant models [28]. rs1537373 is situated in a DNase I hypersensitive region, indicative of active transcription. Based on dual luciferase reporter assays, an electrophoretic mobility shift assay (EMSA), and an expression quantitative trait loci (e‐QTL) analysis, Zhu et al. suggested that the rs1537373‐G risk allele exhibited higher enhancer activity, which regulates differential transcription factor binding, thereby downregulating CDKN2B and increasing the risk of PC [28]. The *CDKN2B* gene encodes the p15Ink4b protein, which forms a complex with cyclin‐dependent kinase 4 (CDK4) or CDK6 to inhibit CDK activity, acting as a cell growth regulator and inhibiting the cell cycle. Moreover, CDKN2B silencing was reported to promote the cell cycle and EMT in HCC cells [47]. Previous studies demonstrated that CDKN2B‐AS1 promotes proliferation and cell cycle progression by targeting E2F transcription factor 1 (E2F1) in HCC cells [40]. We also observed that CDKN2B‐AS1 expression correlated with tumour growth in HCC patients, with the CDKN2B‐AS1‐associated pathway in HCC involving E2F targets and the cell cycle. These findings suggest that genetic mutations of CDKN2B‐AS1 rs1537373 may influence tumour growth by modulating CDKN2B expression in HCC. However, the functional role of *CDKN2B‐AS1* rs1537373 in the tumour growth of HCC warrants further investigation. To elucidate the potential functions of CDKN2B‐AS1 in HCC cell lines and explore the underlying mechanisms, we will construct clones containing various genotypes of CDKN2B‐AS1 SNPs.

To our knowledge, this is the first study to examine associations between variants of CDKN2B‐AS1 and clinicopathological features of HCC in a Taiwanese population. Additionally, we identified a prognostic role for CDKN2B‐AS1 and its related pathways in HCC using clinical samples. Our findings suggest that the CDKN2B‐AS1 rs564398 and rs1537373 SNPs might influence the expression of CDKN2B‐AS1 or CDKN2B, thereby promoting HCC progression. Therefore, the CDKN2B‐AS1 rs564398 and rs1537373 polymorphisms could be crucial markers for predicting HCC tumour aggressiveness and prognoses.

## Author Contributions

**Kuan‐Chun Hsueh:** conceptualization (equal), data curation (equal), methodology (equal), writing – original draft (equal), writing – review and editing (equal). **Hsiang‐Lin Lee:** data curation (equal), resources (equal). **Kuo‐Hao Ho:** software (equal). **Lun‐Ching Chang:** software (equal). **Shun‐Fa Yang:** conceptualization (equal), methodology (equal), writing – original draft (equal), writing – review and editing (equal). **Ming‐Hsien Chien:** conceptualization (equal), writing – original draft (equal), writing – review and editing (equal).

## Conflicts of Interest

The authors declare no conflicts of interest.

## Data Availability

The data that support the findings of this study are available from the corresponding author upon reasonable request.
